# Laparoscopic Management of Bouveret's Syndrome after Failed Endoscopic Approach

**DOI:** 10.1155/2019/7067240

**Published:** 2019-04-18

**Authors:** Ariel Nicolas Tchercansky, Guido Luis Busnelli, Matías Mihura, Rafael José Maurette

**Affiliations:** General Surgery Department, British Hospital of Buenos Aires, Argentina

## Abstract

Bouveret's syndrome is a complication of cholelithiasis that presents with gastric outlet obstruction due to an impacted gallstone in the duodenum following cholecystoduodenal fistula. This is a rare presentation of biliary-enteric fistula; therefore, there are no standardized guidelines for the management of this disease. We present a case of a patient with Bouveret's syndrome managed with laparoscopic surgery after an unsuccessful attempt of endoscopic removal.

## 1. Case Report

A 58-year-old female was presented to the emergency department with a 4-day history of nausea and vomiting. The patient had a past medical history for Parkinson's disease and gallstones but denied having any previous abdominal symptoms. The patient referred abdominal distention with intolerance to oral intake and postprandial vomits of nonbilious characteristics, containing undigested food particles. She also complained of upper abdomen discomfort that relieved after vomiting.

Clinical examination revealed blood pressure of 100/80 mmHg and heart rate of 97 bpm. Cardiorespiratory and neurological exam revealed no abnormalities.

Abdominal examination revealed a soft abdomen with normal bowel sounds, mild epigastric tenderness, and no palpable organomegaly.

**Figure 1:**
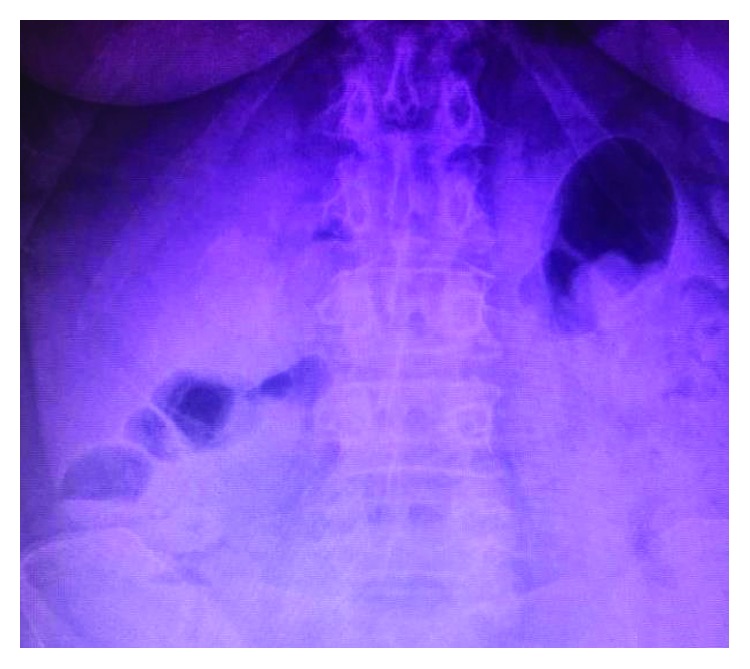
Plain abdominal X-ray: an irregular calcified structure in the right midabdomen.

Plain abdominal X-ray showed an irregular partially rim calcified focus in the right midabdomen and absence of air in the distal bowel (Figure 1). Abdominal ultrasound informed a collapsed gallbladder.

Blood work showed hematocrit of 45%, white count of 7500 cells per mm^3^, urea 48 mg/dl, creatinine 1.02 mg/dl, AST 19 U/l, ALT 16 U/l, alkaline phosphatase 88 U/l, bilirubin 1.4 mg/dl, and amylase 102 U/l.

**Figure 2:**
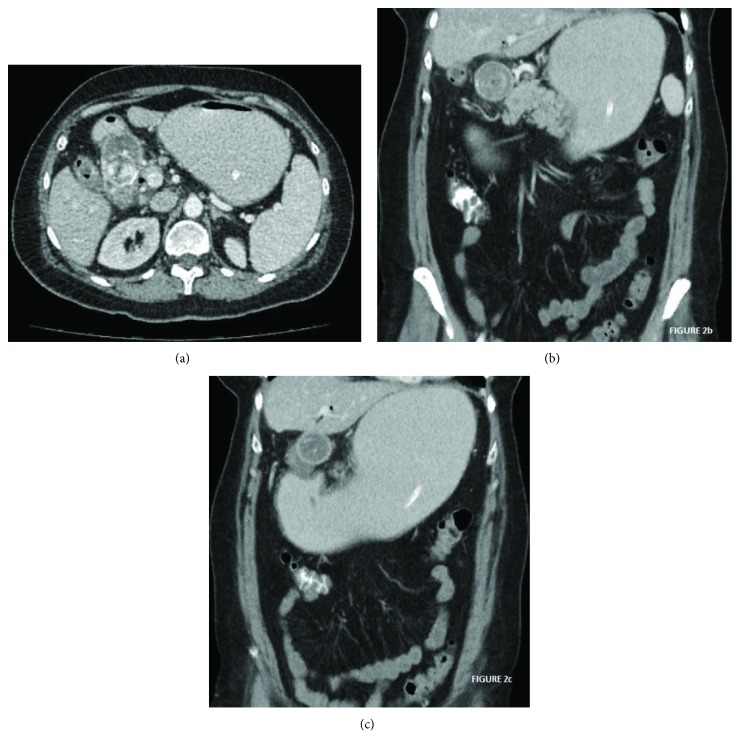
(a) TC axial view: gallstone obstructing the duodenum. (b) TC coronal view: gallstone obstructing the duodenum. Gastric distention. (c) TC coronal view: alteration of the planes surrounding the gallstone and limited pneumobilia.

Initial measures were resuscitation with fluids and gastric decompression with a nasogastric tube. Computed tomography of the abdomen revealed a multilithiasic gallbladder with alteration of the surrounding fatty tissue, pneumobilia, gastric distention, and a 45 mm × 32 mm calcic stone located in the duodenal bulb (Figure 2).

We performed an upper endoscopy identifying an obstructing 4 cm stone in the duodenal bulb. Laser and mechanical lithotripsy were attempted using a Holmium probe and a Dormia basket achieving partial fragmentation of the stone (Video 1), but due to failure of progression, we decided to conclude the procedure and switch to a laparoscopic approach (Video 2).

Two 12 mm and two 5 mm ports were used, all of them in the upper abdomen. Omental adhesions to the gallbladder were lysed exposing the cholecystoduodenal fistula. A decision was made not to treat the fistula due to the high risk of complications. A longitudinal gastric antrotomy was made with ultrasonic shears (harmonic), revealing a partially fragmented stone positioned in the duodenal bulb. The multiple fragments were extracted through the antrotomy with laparoscopic forceps.

The antrotomy was closed with a longitudinal single layer uninterrupted 3/0 absorbable barbed suture. We performed an intraoperative upper endoscopy in order to rule out any air leaks and confirm adequate passage of the scope through the second and third duodenal portions. Finally, the stone fragments were extracted in a retrieval bag, and a Jackson-Pratt drain was placed in the parieto-hepatic recess. Wounds were closed with 2/0 vicryl and 4/0 monocryl.

**Figure 3:**
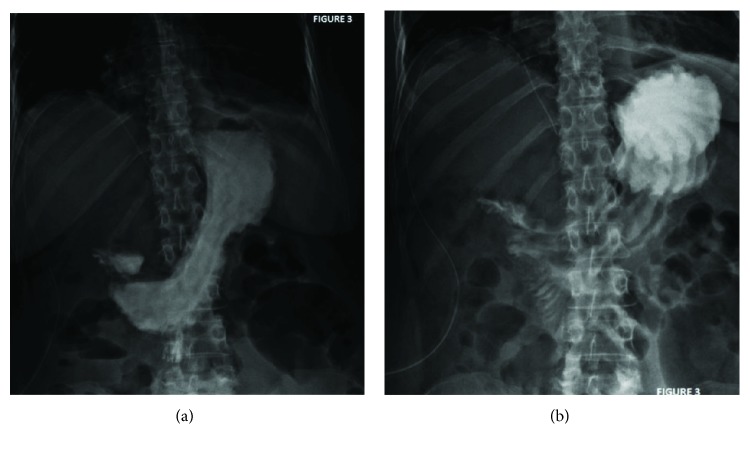
Fluoroscopic study: progression of the contrast through the duodenum without any leaks.

The patient was extubated after surgery and transferred to a general ward. On postoperative day (POD) 4, we performed a fluoroscopic contrast study showing no leaks or obstructions to the passage of the contrast solution (Figure 3). Therefore, the patient initiated liquid oral intake with good tolerance and progressed to a soft diet on POD 5.

The patient was discharged home on POD 12 without any major complications other than an epigastric wound infection that required drainage and oral antibiotics. Outpatient visit one week after discharge revealed adequate oral intake without vomiting or pain. Evaluation over the following months ruled out any complications or recurrence of symptoms.

## 2. Discussion

Common complications associated with cholelithiasis include acute cholecystitis, choledocolithiasis, and pancreatitis, while biliary fistula and gallbladder cancer are uncommon manifestations of long-term gallstone disease. Bouveret's syndrome is a rare complication of cholelithiasis that presents with gastric outlet obstruction due to an impacted gallstone in the duodenum following cholecystoduodenal fistula [1].

Cholecysto-bowel fistula's physiopathology includes chronic inflammation of the gallbladder, firm adhesions to the bowel wall, and an impaired blood supply and venous drainage. This associated with the compression of the gallstone against the gallbladder wall results in necrosis, fistula formation, and subsequent passage of the stone to the bowel developing a gallstone ileus [2, 3]. The development of the fistula depends on which organ is affected, being the cholecystoduodenal fistula the most common one (75% of all cholecystoenteric fistulas), followed by cholecystocolonic (8-26.5%) and least commonly cholecystogastric fistulas [4].

Bouveret's syndrome was first described by Beaussier in 1770 and subsequently named after the French physician Léon Bouveret, who published two cases in 1896 [5]. The disease usually presents as an epigastric colicky pain with nausea and vomiting in older women with a history of cholelithiasis [6]. A review of 128 cases of Bouveret's syndrome showed that most patients present with abdominal pain (71%), nausea, and vomiting (87%) [7].

These patients are usually screened with an abdominal X-ray. Some of the radiologic findings in these patients are pneumobilia (39% of the cases), ectopic gallstone (38%), and distended stomach or bowel (23%) [6]. These combinations of radiological signs are known as the Rigler's triad, which is not pathognomonic of Bouveret's syndrome but suggestive of gallstone ileus.

Most cases present with only one impacted stone, and its removal can be attempted endoscopically with limited success (9%). Usually a mechanical laser or extracorporeal shock wave lithotripsy combined with snares, baskets, and grasping forceps are used; however, most cases require surgery because of the size of the stone [8].

In this scenario, treatment can be aimed at either removing the stone in order to solve the obstruction, removing the gallbladder, and repairing the fistula in a one-step surgery or removing the stone and differing the cholecystectomy and repair of the fistula for a second attempt [9]. Furthermore, the extraction of the stone could be attempted in three different ways: endoscopically, laparoscopically, or via open surgery with the already known benefits of minimally invasive surgery.

The advantages of the one-stage approach include avoiding the necessity of future procedures and the risk of developing cholecystitis, recurrent gallstone ileus, cholangitis, cholangiocarcinoma, and gallbladder cancer, developing in 5-17% of the cases [10]. The incidence of cholangiocarcinoma is 15 times higher in patients with cholecystoenteric fistula with respect to the general population [11]. The largest review article to date of 1001 reported cases of gallstone ileus demonstrated recurrent gallstone ileus in only 6% of patients undergoing an enterolithotomy alone (80% of patients), with an overall rate of 4.7%. The reported mortality rate of the one-stage procedure was 16.9% compared to 11.7% for enterolithotomy alone [12]. Furthermore, the spontaneous closure of the fistulous tract is reported in 50% of the cases [13]. The morbimortality rates of the one-stage procedure can be partially explained not only because of the significant inflammatory process in the right upper quadrant and the presence of edematous duodenal mucosa that make the duodenal defect difficult to repair in the one-stage procedure but also because of the exposure of the patient to a prolonged surgery. Despite this, there is no convincing data that differentiate the outcomes between the one-stage approach vs. the two-stage approach [14].

Considering this, we preferred the two-step over the one-step approach and opted for an initial endoscopic attempt, reserving the laparoscopic approach in case of failure. We believe one-stage procedure should be restricted to relatively young patients in good overall condition.

## 3. Conclusions

Bouveret's syndrome is a rare condition that should be considered in elderly patients with a history of chronic cholelithiasis, epigastric pain, and vomiting.

Surgical options include a combination of enterolithotomy plus cholecystectomy and fistula repair in a one- or two-stage approach.

Endoscopic treatment should be considered as a first attempt, despite the low success rate reported in the literature. If endoscopic treatment fails, surgical treatment should be carried out.

This case report suggests that a laparoscopic approach may represent a valid therapeutic option after unsuccessful endoscopic treatment.

## Supplementary Materials

Supplementary 1 Video 1: endoscopic approach.

Supplementary 2 Video 2: laparoscopic approach: antrotomy and stone extraction.

## References

[1] Leblanc K. A., Barr L. H., Rush B. M. (1983). Spontaneous biliary enteric fistulas. *Southern Medical Journal*.

[2] Langhorst J., Schumacher B., Deselaers T., Neuhaus H. (2000). Successful endoscopic therapy of a gastric outlet obstruction due to a gallstone with intracorporeal laser lithotripsy: a case of Bouveret’s syndrome. *Gastrointestinal Endoscopy*.

[3] Pickhardt P. J., Friedland J. A., Hruza D. S., Fisher A. J. (2003). CT, MR cholangiopancreatography, and endoscopy findings in Bouveret’s Syndrome. *American Journal of Roentgenology*.

[4] Hajjar R., Létourneau A., Henri M. (2018). Cholecystocolonic fistula with a giant colonic gallstone: the mainstay of treatment in an acute setting. *Journal of Surgical Case Reports*.

[5] Bouveret L. (1896). Sténose du pylore adhérent a la vésicule. *Revue Medicale Paris*.

[6] Shah S. K., Walker P. A., Fischer U. M., Karanjawala B. E., Khan S. A. (2013). Bouveret syndrome. *Journal of Gastrointestinal Surgery*.

[7] Cappell M. S., Davis M. (2006). Characterization of Bouveret’s syndrome: a comprehensive review of 128 cases. *The American Journal of Gastroenterology*.

[8] Lowe A. S., Stephenson S., Kay C. L., May J. (2005). Duodenal obstruction by gallstones (Bouveret’s syndrome): a review of the literature. *Endoscopy*.

[9] Fancellu A., Niolu P., Scanu A. M., Feo C. F., Ginesu G. C., Barmina M. L. (2010). A rare variant of gallstone ileus: Bouveret’s syndrome. *Journal of Gastrointestinal Surgery*.

[10] Iñíguez A., Butte J. M., Zúñiga J. M., Crovari F., Llanos O. (2008). Síndrome de Bouveret. Resolución endóscopica y quirúrgica de cuatro casos clínicos. *Revista Médica de Chile*.

[11] Donati M., Cardì F., Brancato G., Calò P., Donati A. (2010). The surgical treatment of a rare complication: gallstone ileus. *Annali Italiani di Chirurgia*.

[12] Reisner R. M., Cohen J. R. (1994). Gallstone ileus: a review of 1001 reported cases. *The American Surgeon*.

[13] Clavien P. A., Richon J., Burgan S., Rohner A. (1990). Gallstone ileus. *British Journal of Surgery*.

[14] Khan A. Z., Escofet X., Miles W. F. A., Singh K. K. (2002). Lessons to be learned: a case study approach. *Journal of the Royal Society for the Promotion of Health*.

